# Corrigendum: Mechanisms Underlying Serotonergic Excitation of Callosal Projection Neurons in the Mouse Medial Prefrontal Cortex

**DOI:** 10.3389/fncir.2019.00023

**Published:** 2019-04-09

**Authors:** Emily K. Stephens, Arielle L. Baker, Allan T. Gulledge

**Affiliations:** Department of Molecular and Systems Biology, Geisel School of Medicine at Dartmouth College, Hanover, NH, United States

**Keywords:** serotonin, cortex, pyramidal neuron, 5-HT2A receptor, calcium, M-current, K_v_7 channels, afterdepolarization

In the original article, there was an error. The original text wrongly suggested that one of our manipulations increased the driving force for potassium by “six-fold”. Instead, while the amount of potassium was lowered six-fold (from 3 mM to 0.5 mM), the driving force for potassium, as measured at the action potential threshold, was approximately doubled.

A correction has been made to the ***Results***, subsection ***Role of M-current in Serotonergic*** ***Excitation***, paragraph three:

“The results above suggest that 5-HT acts via at least three distinct mechanisms (K_V_7 suppression, the ADP conductance, and a calcium-sensitive calcium conductance) to enhance the excitability of COM neurons. To test whether M-current is the dominant potassium conductance contributing to serotonergic excitation, we enhanced the driving force for potassium by lowering the external potassium concentration ([K^+^]_o_) six-fold to 0.5 mM (replaced with equimolar sodium; Figure 7). By increasing the driving force for potassium, this manipulation will enhance the impact of M-current suppression by 5-HT, but will also act to reduce the net current through potassium-permeable non-specific cation conductances. In neurons recorded with control intracellular solution, lowering [K^+^]_o_ revealed a brief inhibition occurring immediately after 5-HT application that was absent in control conditions (Figures 7A,C); these inhibitory responses are likely G_q_-driven hyperpolarizations (mediated by SK-type potassium channels) that occur regularly in pyramidal neurons following M1 muscarinic receptor activation (Gulledge et al., 2009), but which are only rarely observed in response to 5-HT in control conditions. Lowering [K^+^]_o_ enhanced this early potassium conductance, and reduced the magnitude of serotonergic excitation by 31 ± 9% (*n* = 10, paired). In control conditions (e.g., 3 mM [K^+^]_o_), 5-HT generated peak responses of 82 ± 14% with integrals of 157 ± 44 Hz•s. After reducing extracellular potassium to 0.5 mM, peak excitation was 61 ± 15% (*p* = 0.003 relative to control conditions) with integrals of 117 ± 47 Hz•s (*p* = 0.057, Figure 7D). Because the larger driving force for potassium is expected to increase 5-HT excitation by enhancing the contribution of M-current suppression, the observed reductions in response magnitudes and integrals suggest the participation of potassium-permeable non-specific cation conductances, such as the ADP conductance (Haj-Dahmane and Andrade, 1998).”

The authors apologize for this error and state that this correction does not change the scientific conclusions of the article in any way. The original article has been updated.
